# Bone mesenchymal stem cell extracellular vesicles delivered miR let-7-5p alleviate endothelial glycocalyx degradation and leakage via targeting ABL2

**DOI:** 10.1186/s12964-023-01229-7

**Published:** 2023-08-16

**Authors:** Zhe Li, Yuqing Xu, Shiyue Lu, Yuan Gao, Yuxiao Deng

**Affiliations:** Department of Critical Care Medicine, Renji Hospital, School of Medicine, Shanghai Jiao Tong University, No. 160, Pujian Road, Pudong New District, Shanghai, 200120 China

**Keywords:** Endothelial glycocalyx, Mesenchymal stem cells, Extracellular vesicles, ABL2, Let-7

## Abstract

**Background:**

Endothelial glycocalyx (EG) is an active player and treatment target in inflammatory-related vascular leakage. The bone marrow mesenchymal stem cells (bMSCs) are promising potential treatments for leakage; however, the therapeutic effect and mechanism of bMSC on EG degradation needs to be elucidated.

**Methods:**

EG degradation and leakage were evaluated in both lipopolysaccharide (LPS)-induced mice ear vascular leakage model and LPS-stimulated human umbilical vein endothelial cells (HUVECs) model treated with bMSCs. Extracellular vesicles (EVs) were extracted from bMSCs and the containing microRNA profile was analyzed. EV and miR let-7-5p were inhibited to determine their function in the therapeutic process. The ABL2 gene was knockdown in HUVECs to verify its role as a therapeutic target in EG degradation.

**Results:**

bMSCs treatment could alleviate LPS-induced EG degradation and leakage in vivo and in vitro, whereas EVs/let-7-5p-deficient bMSCs were insufficient to reduce EG degradation. LPS down-regulated the expression of let-7-5p while upregulated endothelial expression of ABL2 in HUVECs and induced EG degradation and leakage. bMSC-EVs uptaken by HUVECs could deliver let-7-5p targeting endothelial ABL2, which suppressed the activation of downstream p38MAPK and IL-6, IL-1β levels, and thus reversed LPS-induced EG degradation and leakage.

**Conclusion:**

bMCSs alleviate LPS-induced EG degradation and leakage through EV delivery of miR let-7-5p targeting endothelial ABL2.

**Supplementary Information:**

The online version contains supplementary material available at 10.1186/s12964-023-01229-7.

## Abstract (plain language summary)

**Background **Inflammation-related Endothelial vascular leakage (EVL) is associated with poor clinical prognosis. Endothelial glycocalyx (EG) is a novel therapeutic target for EVL. bMSCs (Bone Mesenchymal Stem Cells) are potential therapies for EVL, but the effect of bMSCs on EG has not been investigated.

**Significance **bMSCs alleviating EG degradation and leakage was firstly clarified in our LPS-induced vascular leakage mice model. Histology and electrophysiology experiments validated that bMSCs achieve therapeutic effects through paracrine extracellular vesicles (EVs). EV-delivered MicroRNA sequencing revealed that miR let-7-5p down-regulated endothelial ABL2/p38MAPK-related inflammation activation. The bMSC-EV delivered let-7-5p was proved as an effective element in alleviating inflammation-related EG degradation and leakage, providing an executable approach for bMSCs to treat EVL.

Video Abstract

**Supplementary Information:**

The online version contains supplementary material available at 10.1186/s12964-023-01229-7.

## Background

Endothelial vascular leakage (EVL) is the hallmark of a variety of life-threatening conditions including sepsis, acute respiratory distress syndrome (ARDS), and capillary leakage syndrome, and contributes to the morbidity and mortality of critically ill patients [1–3]. Despite the serious adverse clinical outcome associated with this pathologic process, no treatment is currently available to reverse EVL [4–6].

The changes in endothelium structure caused by the release of inflammatory mediators during these diseases are believed to play a role in EVL [7]. The endothelial glycocalyx (EG), a covering surface consisting of glycoproteins and proteoglycans in the endothelial vascular lumen, is essential for homeostasis in the endothelial cells (ECs) [8]. In animal studies, a decrease in EG thickness and an increase in plasma concentration of EG fraction syndecan-1(the core protein of EG proteoglycan) usually occurred 2 h after the initial injury [9]. Under the circumstance of endothelial inflammatory activation, the endothelial glycocalyx seemed to be an early victim and would be degraded by MMP-9 (an activated degrading enzyme during inflammation), which leads to loss of vascular tone while increased the permeability [10, 11]. What’s more, the serum syndecan-1 level has been proven to be the most potential biomarker for organ dysfunctions in experimental and clinical studies [12, 13]. Current views believed that preventing EG degradation is a potential and effective therapeutic approach for EVL [14]. Nevertheless, despite advancements in our understanding of molecular mechanisms underlying inflammation-related EG degradation, there was still no effective treatment to directly counteract the degradation of EG in clinical studies [1, 15]. Thus, finding an applicable treatment to prevent EG degradation is essential for the clinical treatment of EVL.

Bone Marrow Mesenchymal Stem Cells (bMSCs), known for their immunomodulatory capacity, was regarded as a potential treatment for endothelial injury including EVL [16]. Cell therapy by bMSCs has been documented to be capable of alleviating lipopolysaccharide (LPS)-induced inflammation and acute lung injury in our preclinical studies, and it also was applied in several human clinical studies for sepsis and ARDS [17–20]. However, in these studies, the therapeutic efficiency of bMSCs requires improvements [21, 22]. The concrete mechanism of how bMSCs treated EVL has not been fully understood, which prevents the clinical development of bMSC cell therapy.

Recent studies suggest that the therapeutic benefits of bMSCs might be mediated by the paracrine effect through extracellular vesicles (EVs) other than the conventional repairment [23, 24]. bMSC-EVs not only have similar therapeutic effects as bMSCs, but also offer additional advantages, including low immunogenicity and convenience for storage. EVs regulate intercellular communication and biological function by delivering internal proteins, lipids, and nucleic acids [23]. MicroRNAs (miRNAs) delivered by bMSC-EVs could be uptaken by targeting cells and be involved in the regulation of gene expression and related signaling pathways [25]. For example, cell-to-cell transfer of miR-125a from MSC-EVs to ECs was proven to promote angiogenesis [26]. Moreover, it was reported that bMSC-EVs could improve LPS-induced pulmonary edema by inhibiting microvascular inflammatory cytokine levels [27, 28]. However, the mechanism of how miRNA delivered by bMSC-EVs regulate EG degradation and leakage as well as the involved target gene remain to be explored.

This study aimed to elucidate the therapeutic effect of bMSCs on LPS-induced EG degradation and leakage and to determine if and how miRNAs delivered by bMSC-EVs play a role in this process.

## Methods

### Ethics statement and animals

Male BALB/c mice (6–8 weeks, 20–24 g) were purchased from the Institute of Laboratory Animal Science, Chinese Academy of Medical Sciences (Shanghai, China). The mice were housed at a constant temperature (24 °C ± 2 °C) and humidity (50%–60%) under a 12-h light/dark cycle with free access to standard food and water. Animal-related experimental procedures were approved by the Animal Care and Use Committee of Shanghai Jiao Tong University (No.20220126). The study was reported as per the Animal Research: Reporting of In Vivo Experiments (ARRIVE) guideline.

### LPS-induced ear vascular leakage mice model and procedures

Based on our previous study of an early (6 h) ear leakage model in LPS-stimulated mice [29], male BALB/c mice aged 6–8 weeks (20–24 g) were anesthetized (RC2 Rodent Anesthesia System) using an isoflurane pill 24 h after ear hair removal. Then, both the left and right ears were pricked using an insulin syringe (27 G needle). For the sham and bMSC groups, both the left and right ears were smeared with 20 μL normal saline. As for the LPS and LPS + bMSC groups, the left ear was smeared with normal saline and the right ear was smeared with 20 μL LPS (L2630; Sigma-Aldrich, USA) at a concentration of 125 μg/μL. After 2 h, the mice in the sham and LPS groups were slowly injected with 100 μL phosphate-buffered saline (PBS, G4202, Servicebio, China) via the intravenous tail, whereas the mice in the bMSC and LPS + bMSC groups were injected with primary bMSCs (2 × 10^7^ cells) resuspended in an equal amount of PBS. At 6 h after LPS administration, the mice were sacrificed, ear tissues were harvested, and serum samples were collected for further studies.

### Ear vascular leakage evaluation

2% Evans blue (EB, 100 μL, E2129, Sigma-Aldrich, USA) was slowly injected into the mice intravenous tail at 2 h before perfusion and sacrifice. The mice ear was cut into an 8-mm disk, after weight and thickness measurement, each ear disk was chopped and immersed in formamide solution (A100606, SangonBio, China) at 50 °C for 72 h. The supernatant was centrifuged at 15,000 g (10 min) for EB absorbance measurement by spectrophotometer (Tristar^2^ S LB 942, Berthold; wavelength, 630 nm). The swelling ratio, thickening ratio, and extravascular EB leakage ratio were calculated, and the left ear was used as a reference.

### Cell culture and modeling

Commercialized primary human bMSCs (HUXMA-01001, Cyagen, China) were cultured in a growth medium for human MSCs (HUXMA-90011, Cyagen, China), and cells at passages 3–6 were used for experiments. Human umbilical vein endothelial cell lines (HUVECs, Cell Applications, USA) were cultured in DMEM/F12 medium (Gibco, USA) in an environment of the humidified atmosphere at 37 °C with 5% CO_2_. All culture mediums contained 10% EV-free FBS (Exo-FBS-50a-1, BI, USA) with 1% penicillin–streptomycin (Gibco, USA). For co-culture experiments, bMSCs were seeded on Transwell inserts in six-well plates (3450, Corning, USA) and HUVECs were seeded in six-well plates. When HUVECs attained 80% confluency, bMSCs and HUVECs were co-cultured in a refreshing medium. HUVECs were stimulated with LPS (1 μg/mL) for 6 h and then were collected for related experiments.

### Isolation, identification, and labeling of EVs from bMSCs

The bMSC culture supernatant was collected and sequential centrifuged at 500 × g (10 min), 3,000 × g (20 min), and 10,000 × g (60 min) to remove impurities and then ultracentrifuged at 120,000 × g for 70 min (XPN-100, Beckman, USA) to precipitate the EVs. The purified EVs were placed on a carbon-coated copper mesh (90 s) and were stained with uranyl acetate dye solution (30 s) and then observed under a transmission electron microscope (Hitachi H-7500). Nanosight NS5300 (Malvern Panalytica, UK) was used to measure the particle size distribution based on a nanoparticle tracking analysis (NTA). EV surface marker protein levels were further measured by WB. The purified EVs were labeled using PKH-67 membrane dye (Sigma–Aldrich, USA) under the manufacturer’s protocol.

### Preparation of bMSC medium, EV-deficient bMSC, and EV-deficient bMSC medium

For the preparation of the bMSC culture medium (bMSC med), the EV-free medium was replaced at 60% cell confluence and collected after 48 h. The EV secretion inhibitor (GW4869,10 μM, Selleck, USA) was administrated to inhibit EV production [30] for the preparation of EV-deficient bMSC (bMSC-GW4869) and EV-deficient bMSC medium (bMSC-GW4869 med).

### Treatment of bMSCs with miR let-7-5p inhibition

miR let-7-5p inhibitor (-CUACUACCUCA-, Sangon Co., Ltd., China) was transfected to the bMSCs using Lipofectamine® 8000 (Invitrogen, USA) according to the manufacturer’s protocol. The efficiency was confirmed by qRT-PCR of let-7a-5p, let-7b-5p, and let-7i-5p expression.

### Treatment of HUVECs with ABL2 knockdown

To knock down ABL2, HUVECs were infected with lentiviral ABL2-shRNA. The 293 T cells were transfected with ABL2-shRNA plasmids (LncBio, China) using Lipofectamine 2000 (Invitrogen, USA) according to the manufacturer’s instructions.

The efficiency of the ABL2 knockdown in HUVECs was confirmed by ABL-2 protein and gene expression.

### miRNA sequences of bMSC-EVs

bMSC-EV containing miRNA was extracted using the MagZol Reagent (Magentec, USA) according to the manufacturer’s protocol. Sequencing analysis of miRNAs was performed by HiSeq 2500 (Illumina, USA). After data filtering, the genome-wide distribution profiles were obtained by aligning clean reads to the reference genome using Burrows-Wheeler Alignment Tool. The miRNAs were identified using miRdeep2 based on the miRBase version 22, and the expression level and clustering were further calculated. Heatmap R package was used for displaying miRNA expression. The target genes of specific miRNAs were predicted using the TargetScan, miRDB, miRTarBase, and miRwalk databases. Kyoto Encyclopedia of Genes and Genomes (KEGG) and Gene Ontology (GO) databases were used for functional analysis.

### Transmission electron microscopy (TEM)

Fresh right ear tissues were trimmed into 1-mm^3^ sections and quickly immersed into glutaral at 4 °C for 4 h. After permeation, dehydration, and embedding, samples were mounted on the carbon-coated copper mesh and observed under a transmission electron microscope (Talos L120C, Thermo Fisher, USA).

### Enzyme-linked immunosorbent assay (ELISA)

The mice's right ear tissues were triturated with PBS and centrifugated. The protein concentrations of IL-6 and IL-1β (mouse IL-6 IL-1β ELISA kit, Liankebio, China) were quantified according to the manufacturer’s instructions.

### Hematoxylin and eosin (H&E) staining

The right ear tissues were fixed with 4% paraformaldehyde, embedded in paraffin, and sectioned into 5-μm slices. The paraffin sections were stained using H&E solution (Beyotime Biotechnology, China) and observed under an optical microscope (IX73, Olympus, Japan).

### Immunohistochemistry (IHC) staining

The right ear tissues were microwaved in 10-mm citrate buffer (pH 6.0) and stained with primary antibodies using the enzyme complex method. After incubation with the secondary antibodies and streptavidin solution, color development was performed using 3,3-diaminobenzidine tetrahydrochloride. Sections were counterstained using Gill-2 hematoxylin (Thermo Fisher, USA) and dehydrated using ethanol and xylene, and then observed under an optical microscope (IX73, Olympus, Japan).

### Total protein extraction and BCA

 Tissues, cells, or EVs were lysed and extracted by RIPA lysis buffer (Epizyme, China) for 15 min on ice. After centrifugation at 15,000 × g for 15 min at 4 °C, the concentration of protein supernatant was determined using the BCA protein assay kit (Epizyme, China).

### Western blot analysis

The extracted protein was separated using SDS-PAGE and transferred to the polyvinylidene fluoride membrane. After blocking, the membrane was probed with primary antibodies (Table S1) at 4 °C overnight and incubated with horse-radish peroxidase-conjugated secondary antibody (Beyotime, China) at room temperature for 1 h. The immunoreactive proteins were detected using an enhanced chemiluminescent kit (Vazyme, China) and analyzed using ImageLab software (Bio–Rad, USA).

### Immunofluorescence (IF) staining

The right ear tissue sections and the HUVECs climbing slices were incubated with primary antibodies overnight at 4 °C. And then rinsed with secondary antibodies (Table S1) for 30 min at room temperature and sealed with DAPI staining solution (Beyotime Bio, China). The sections were observed under a fluorescence microscope (IX73, Olympus, Japan) or confocal microscope (FluoView FV3000, Olympus, Japan).

### Quantitative real-time-PCR (qRT–PCR)

The total RNA of HUVECs was extracted using a TRIzol reagent (Invitrogen, USA). RNA was reversed using miRNA 1st Strand cDNA Synthesis Kit (by stem-loop) (Vazyme, China). qRT-PCR was performed using ChamQ SYBR Color qPCR Master Mix (Vazyme, China) on an iq5 real-time PCR detection system (Bio-Rad Laboratories Inc., USA). mRNA expression was normalized to that of Actin or gapdh, and miRNA expression was normalized to that of U6 and analyzed using the 2^−ΔΔCt^ method. All primers (Table S2) were manufactured by Sangon Biotech, Co., Ltd. (China).

### Luciferase reporter assay

The 3′-UTR sequences containing wild-type and mutant let-7-5p binding sites were synthesized and cloned into pmirGLO vectors (Biogene Co., Ltd. Shanghai).Let-7-5p transfected HUVECs were seeded in 24-well plates and incubated with pmirglo-ABL2-3′UTR-Wt or pmirglo-ABL2-3′ UTR-Mut for 24 h. The luciferase activity was detected using a Dual Luciferase Assay Kit (Promega, USA) according to the manufacturer’s protocol.

### Transendothelial electrical resistance (TEER)

TEER was used to assess the EVL in vitro. HUVECs were seeded on Transwell inserts of 12-well plates (3401, Corning, USA) with the corresponding treatments. TEER was measured every 60 min by inserting electrodes (EVOM^2^, WPI, USA) into the medium of the upper and lower chambers. The baseline resistance was used as the reference.

### Statistical analysis

The numerical data were represented as mean ± SEM. GraphPad Prism 9 (GraphPad Software Inc, CA) was used for data analysis. Statistic analysis was performed using Unpaired t-test and Mann–Whitney U test according to normality test for comparisons between the two groups and one-way or two-way ANOVA for multiple comparisons. *P* < 0.05 was considered statistically significant.

## Results

### The therapeutic effect of bMSCs on LPS-induced EG degradation and leakage in mice

**Fig. 1:**
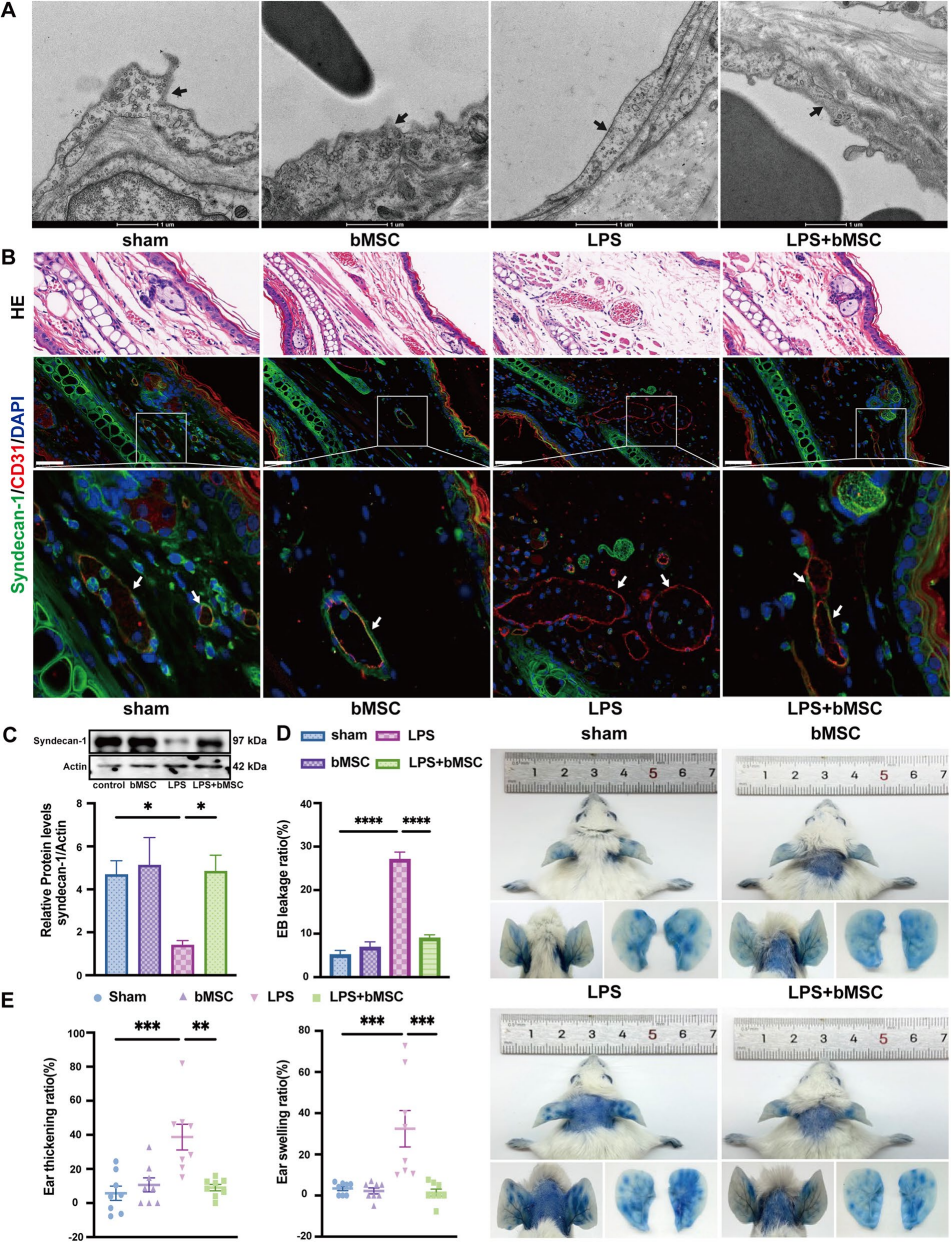
bMSCs alleviate LPS-induced endothelial glycocalyx (EG) degradation and leakage in vivo. **A **Transmission electron microscopy images of EG in mice right ear tissues. Black arrows: the EG layer. Scale bar: 1 μm (*n* = 6). **B **Immunofluorescence images of syndecan-1 (green) and CD31 (red) in vascular endothelium (white arrows) contrast with hematoxylin and eosin staining of the upper or lower adjacent sections in mice right ear tissues; blue: DAPI. Scale bar: 50 μm (*n* = 6). **C **Western blot images and quantitative analysis of the syndecan-1 levels in mice right ear tissues compared to that of Actin (*n* = 6). **D **Ear leakage images displayed with Evans blue (EB) and quantitative analysis of ear EB leakage ratios (*n* = 8). **E **Quantitative analysis of ear swelling and thickening ratio (*n* = 8). EB leakage ratio = (*aEB*_*r*_ − *aEB*_*l*_)/*t*_*l*_ × 100%; *aEB*: EB absorbance; Swelling ratio = (*w*_*r*_ − *w*_*l*_)/*w*_*l*_ × 100%; *w:* weight. Thickening ratio = (*t*_*r*_ − *t*_*l*_)/*t*_*l*_ × 100%; *t*: thickness. *r*: right ear; and *l*: left ear. All results were obtained at 6 h after LPS administration. Values are presented as mean ± sems. Statistical analysis: *p < 0.05, **p < 0.01, ***p < 0.001, ****p < 0.0001

In order to assess the therapeutic efficacy of bMSCs on EG degradation and leakage in vivo, we constructed a mice ear vascular leakage model by pricking and smearing LPS for 6 h, and the scheme for the animal study was shown in Figure S1. According to the TEM imaging, the LPS group resulted in the disappearance of the glycocalyx layer in the vascular lumen compared with the sham group. Treatment with bMSCs ameliorated the destruction of the glycocalyx layer by LPS (Fig. 1A). In accordance with the TEM observations, LPS induced a reduced syndecan-1 fluorescence intensity co-staining with CD31 + on ear vascular ECs (Fig. 1B) and a threefold decrease of the syndecan-1 protein levels in the ear tissue (Fig. 1C) compared with the sham group, which were also blocked by bMSCs treatment. The ear EVL was determined by ear EB leakage, swelling, and thickening. In the LPS group, the EB color was more apparent in the right ear than in the left ear and the EB leakage ratio was threefold higher compared with that in the sham and sham + bMSC groups (Fig. 1D). Similarly, the swelling and thickening ratio was higher in the LPS group and was improved by bMSCs treatment compared with those in the sham group (Fig. 1E). In addition, the increased inflammatory response is also well-accepted as a hallmark of EG degradation and leakage. HE staining showed increased neutrophil infiltration and thickening in the ear in the LPS group compared with that in the sham group, and the phenomenon could be mitigated after bMSCs treatment (Figure S1A). Accordingly, concentrations of proinflammatory cytokines of IL-6 and IL-1β in ear tissue were remarkably elevated in the LPS group than that in the sham group but would be decreased after bMSCs treatment (Figure S1B).

### The therapeutic effect of bMSCs on LPS-induced EG degradation and leakage in HUVECs

**Fig. 2:**
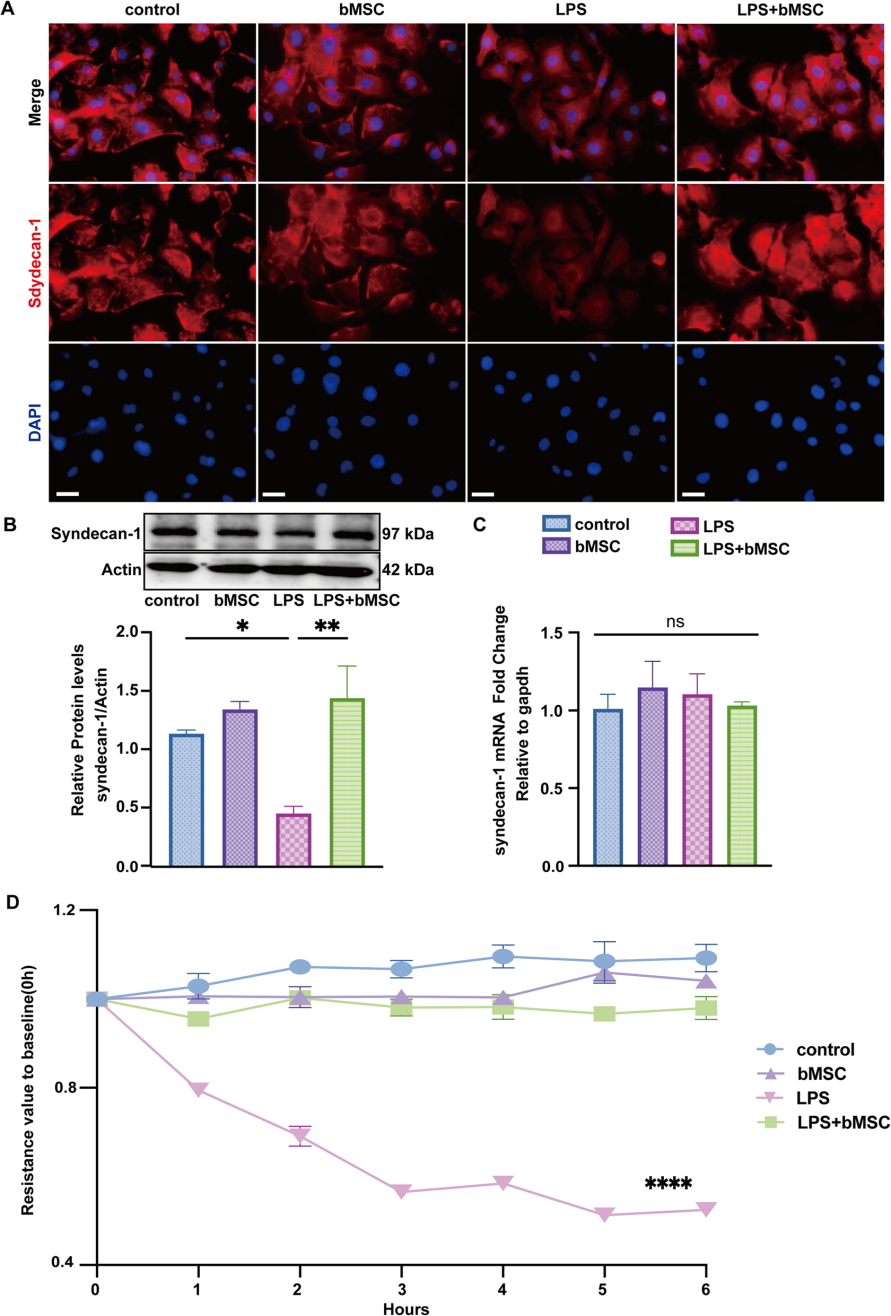
bMSCs alleviate LPS-induced endothelial glycocalyx (EG) degradation and leakage in vitro. **A **Immunofluorescence images of syndecan-1(red) expression in human umbilical vein endothelial cells (HUVECs); blue: DAPI. Scale bar: 50 μm. **B **Western blot images and quantitative analysis of the syndecan-1 levels in HUVECs compared with that of Actin (*n* = 3). **C **qRT-PCR analysis for fold changes of syndecan-1 gene expression to that of gapdh in HUVECs (*n* = 3). **D **Transendothelial resistance value to baseline (0 h) in HUVECs, X-axis: time (h) (*n* = 3). All results were obtained at 6 h after LPS administration. Values are presented as mean ± sems. Statistical analysis: *p < 0.05, **p < 0.01, ***p < 0.001, ****p < 0.0001

HUVECs were co-cultured with bMSCs for 6 h after LPS stimulation to confirm the therapeutic efficacy of bMSCs on ECs. Immunofluorescence staining showed that the expression of syndecan-1 protein (red) located on the cell membrane reduced sharply compared with that of the control group based on the fluorescence intensity, but its expression could be recovered after HUVECs were co-cultured with bMSCs (Fig. 2A), consistent with our Western blot analysis results of syndecan-1 protein levels (Fig. 2B). Our PCR results further showed that neither LPS nor bMSCs treatment would have impacts on the syndecan-1 gene expression (Fig. 2C). We further valued the EVL status with TEER. The resistance value in the LPS group began to decrease after 1 h and maintained only 50% of the initial level after 6 h compared with the control. While it remained stable within 6 h in the LPS + bMSC group as that in the control group (Fig. 2D). These results evidenced the protective effect of bMSC on EG and leakage and suggested the mechanism lay in preventing EG protein degradation rather than regeneration. Additionally, a similar trend was observed for the concentrations of pro-inflammatory IL-6 and IL-1β as in the in vivo results (Figure S1C), supporting our conjecture that bMSCs exert a protective effect by reducing the inflammatory response.

### bMSC-EVs play a key role in the therapeutic effect of bMSCs

bMSC culture medium was obtained to investigate the influence of the paracrine effects on efficacy. LPS induced a twofold decrease in syndecan-1, a twofold increase in MMP-9 (syndecan-1 cleavage enzyme), and a twofold increase in proinflammatory IL-6, and IL-1β protein levels compared with the control group. However, all these variations can be ameliorated after treatment of either bMSCs or bMSC culture medium, and the culture medium could suppress MMP-9 increase more significantly than the bMSCs (Figure S3A). Additionally, neither LPS, bMSCs nor bMSC medium affect syndecan-1 gene expression. Although LPS induced a fourfold increase in MMP-9 gene expression, the trend was suppressed in bMSC or bMSC medium treated groups (Figure S3B). These results indicated that the components in the bMSC culture medium may play a key role in treating LPS-induced EG degradation.

**Fig. 3:**
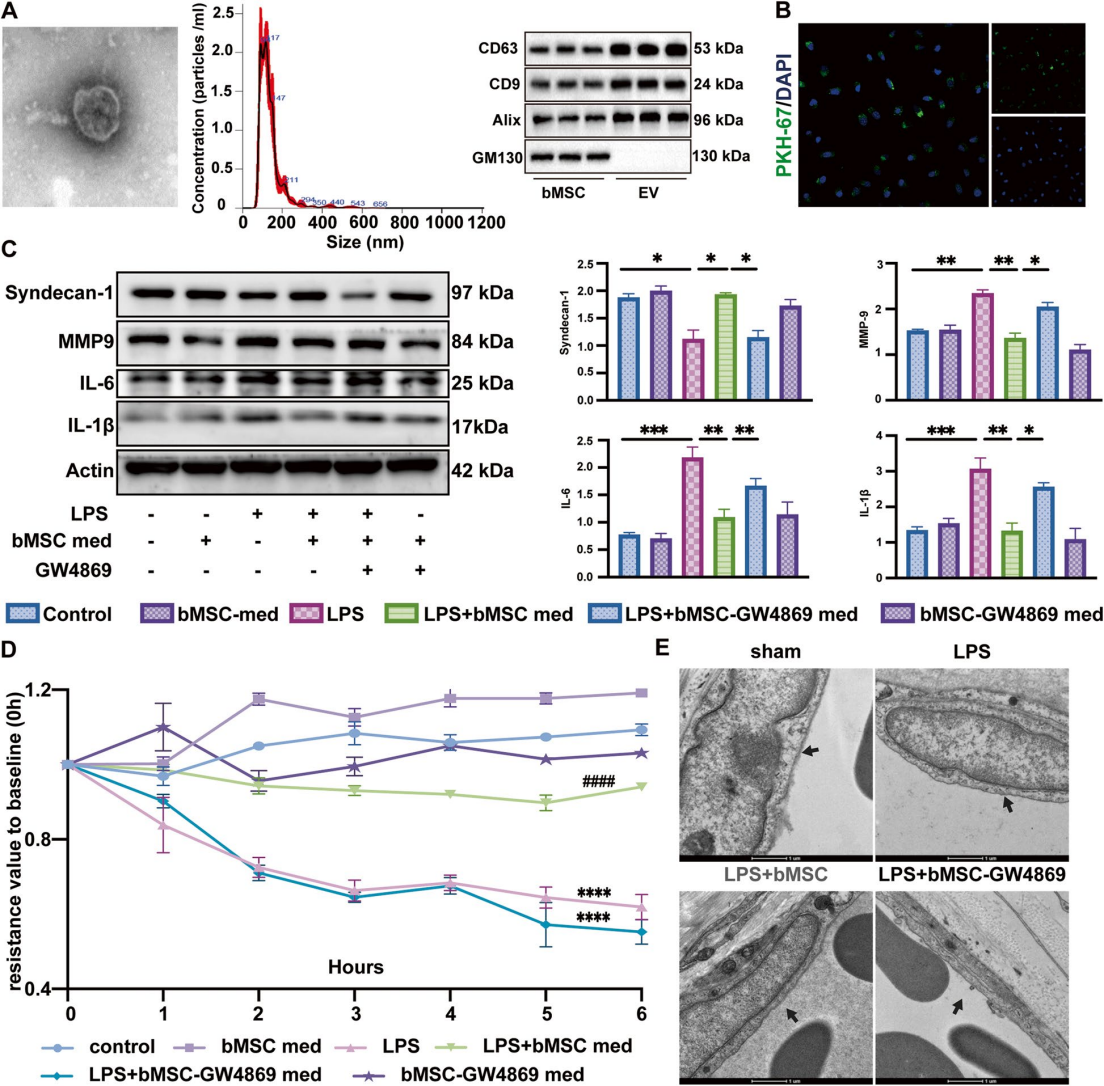
bMSC delivered extracellular vesicles (EVs) mediates the therapeutic effect of bMSCs on endothelial glycocalyx (EG) degradation. **A **Identification of bMSC-EVs: left, transmission electron microscopy image of the EV structure, scare bar: 100 nm; middle, EV size analysis using nanoparticle size analyzer; right, EV markers CD9, CD63, Alix, and GM130 protein images by Western Blot. **B **Confocal microscopy shows the fluorescence of uptake of PKH-67-labeled EVs (green) by HUVECs. Scale bar: 100 μm (*n* = 3). **C **Western blot images and quantitative analysis of syndecan-1, MMP-9, IL-6, and IL-1β protein levels compared to that of Actin in HUVECs treated with bMSC med and bMSC-GW4869 med (*n* = 3). GW4869: EVs inhibitor. **D **Transendothelial resistance value to baseline (0 h) in HUVECs with the same treatment as described in (C) (*n* = 3), ^####^*p* < 0.0001 compared with the LPS group. **E **Transmission electron microscopy images of EG in mice right ear tissues treated with bMSCs and bMSCs-GW4869, black arrows: the EG layer, Scale bar: 1 μm (*n* = 6). All results were obtained at 6 h after LPS administration. Values are presented as mean ± sems; statistical analysis: *p < 0.05, **p < 0.01, ***p < 0.001, ****p < 0.0001

To evidence the uptake of bMSC-EVs by HUVECs in vitro, EVs were isolated from the culture medium of bMSCs by ultracentrifugation. TEM image and nanoparticle size analyzer revealed that MSC-EVs had a cup-shaped morphology with a diameter of 50–150 nm in size, and EVs protein was confirmed positive for EV markers CD9, CD63, Alix while negative for cell debris marker GM130 (Fig. 3A). HUVECs were then cultured with PKH67-labeled bMSC-EVs. Confocal microscopy results showed the labeled bMSC-EVs fluorescence in HUVECs after 6 h (Fig. 3B).

To determine the EVs are exact effector components of bMSCs med on LPS-induced EG degradation, bMSCs were pretreated with EV inhibitor (bMSC-GW4869) and the medium was obtained after the inhibition. A suppression rate of 4000 times in particle concentration and a minimum of 70% fold decrease in positive surface marker protein concentration of EV secretion after GW4869 treatment were identified by Nano Flow Cytometer (NanoFCM) (Figure S3C) and western-blot (Figure S3D), respectively. bMSC-GW4869 med treatment failed to ameliorate the LPS-induced decrease in the syndecan-1 level, the increase in the MMP-9 level, and the increase in proinflammatory IL-6, IL-1β levels (Fig. 3C). bMSC-GW4869 med also diminished the effect of bMSC med on maintaining TEER value after LPS stimulation (Fig. 3D). These results suggest that bMSC-EVs are pivotal effector components for reducing EG degradation and leakage in vivo.

Moreover, we performed in vivo validation with mice treated with bMSCs and bMSC-GW4869. bMSCs without EVs secretion lost the ability to ameliorate LPS-induced glycocalyx layer destruction in mice ears compared with normal bMSCs (Fig. 3E). All over, the above results confirmed that bMSC-EVs play a key role in the treatment of LPS-induced EG degradation.

### Essential role of miR-let-7 in mediating the effects of bMSC-EVs on LPS-induced EG degradation and leakage

**Fig. 4:**
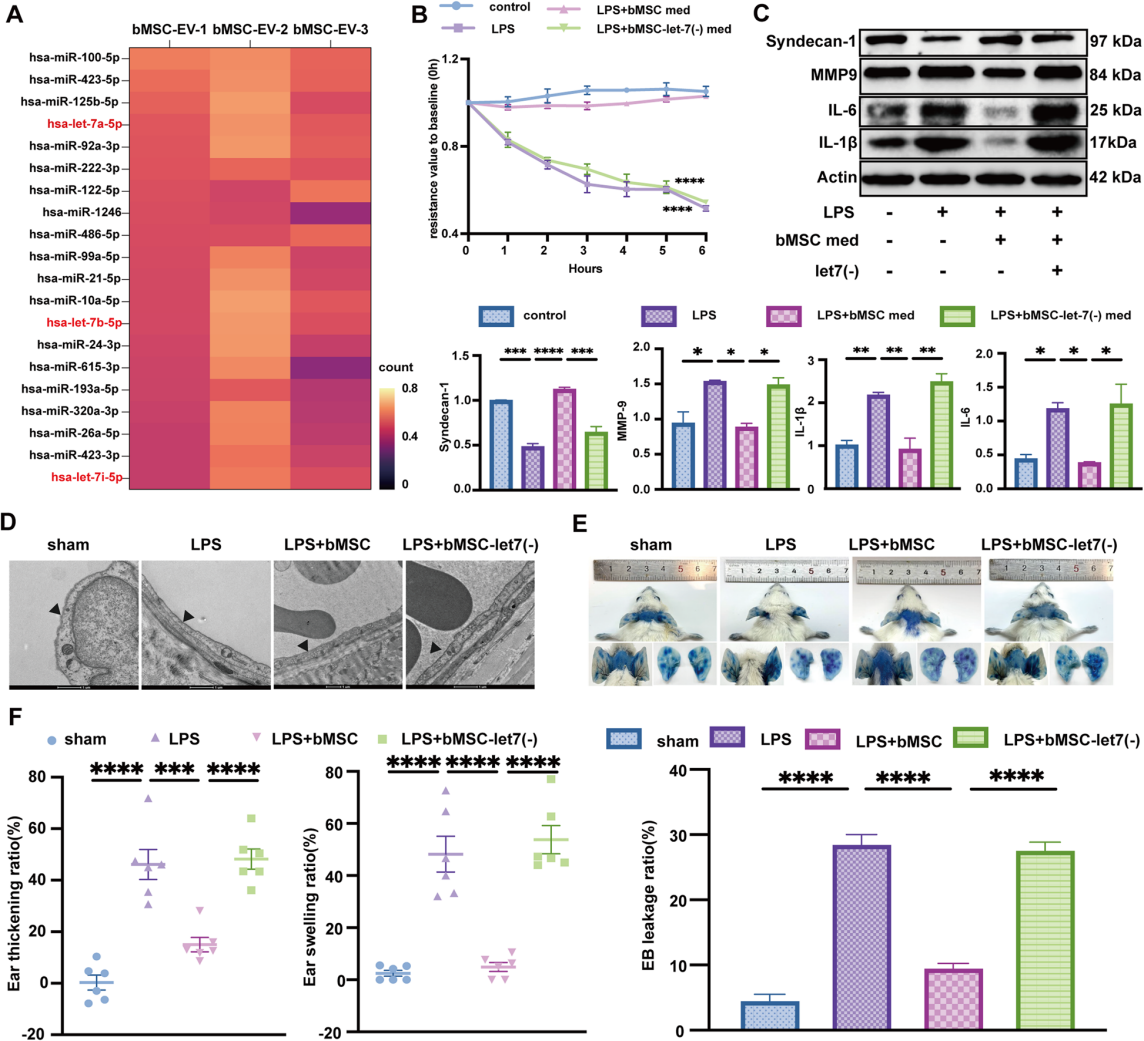
Insufficient inhibition of let-7-deficienct bMSC med on endothelial glycocalyx (EG) degradation and leakage. **A **Heat map of the top 20 miRNA co-expressed in bMSC-EVs (*n* = 3). **B **Transendothelial resistance value to baseline (0 h) in human umbilical vein endothelial cells (HUVECs) treated with normal bMSC med and let-7-deficient bMSC med. X-axis: time (h), (*n* = 3). **C **Western blot images and quantitative analysis of syndecan-1, MMP-9, IL-6, and IL-1β levels compared with that in HUVECs treated as described in (**B**) (*n* = 3). **D **Transmission electron microscopy images of EG in mice right ear tissues treated with normal bMSCs and let-7-deficient bMSCs. black arrows: the EG layer. Scale bar: 1 μm (*n* = 6). **E **Ear leakage images displayed with Evans blue (EB) and quantitative analysis of ear EB leakage ratios treated as described in (**D**) (*n* = 6). **F **Quantitative analysis of ear swelling and thickening ratio (*n* = 6). EB leakage ratio = (*aEB*_*r*_ − *aEB*_*l*_)/*t*_*l*_ × 100%; *aEB*: EB absorbance; Swelling ratio = (*w*_*r*_ − *w*_*l*_)/*w*_*l*_ × 100%; *w:* weight. Thickening ratio = (*t*_*r*_ − *t*_*l*_)/*t*_*l*_ × 100%; *t*: thickness. *r*: right ear; and *l*: left ear. For B, C, D, E, and F results were obtained at 6 h after LPS administration. Values are presented as mean ± sems. Statistical analysis: *p < 0.05, **p < 0.01, ***p < 0.001, ****p < 0.0001

miRNAs delivered by EVs are transferred between cells as a way of intercellular communication. The expression profile of bMSC-EVs delivered miRNA identified three miR-let-7 family members (let-7a-5p, let-7b-5p, and let-7i-5p) among the top 20 highly co-expressed miRNAs (Fig. 4A). It was reported that miR let-7-5p participated in acute and chronic inflammatory processes in diseases such as wound healing and diabetes [31]. Therefore, we focused our study on let-7-5p. To study the essential role of let-7 in bMSC med, a shared let-7 inhibitor sequence was transfected to bMSC (bMSC-let-7[−]) and a two-to-three-fold suppression of let-7a-5p, let-7b-5p, and let-7i-5p expressions was confirmed (Figure S3A). qRT-PCR further showed that LPS down-regulated let-7a-5p, let-7b-5p, and let-7i-5p expression at the level of 3–fourfold change in HUVECs, while treated with bMSC med increased the let-7-5p levels, however, the absence of either EV or let-7-5p in bMSC med failed to increase the let-7-5p levels (Figure S3B), suggesting that EV delivered let-7-5p from the bMSC paracrine to the HUVECs played a regulatory role. Protein levels in the HUVECs after LPS stimulation showed that EVs or let-7-5p inhibition in bMSC med deprives its prevention effect of MMP-9 increase and syndecan-1 decrease at the same degree (Figure S3C). These conform to our speculation that let-7 is the main effector component in bMSC-EVs to suppress EG degradation.

TEER values of HUVECs treated with bMSC-let-7(−) med showed that let-7-5p deficiency deprived the maintained effect of bMSC med on TEER value after LPS stimulation (Fig. 4B), consistent with the protein levels variation of syndecan-1, MMP-9 and proinflammatory IL-6, IL-1β (Fig. 4C). To further verify the major mediate role of let-7-5p in vivo, we treated mice with bMSCs or bMSCs-let-7(−) after LPS administration. Let-7-5p deficiency in bMSCs also failed to ameliorate LPS-induced mice ear EG layer destruction (Fig. 4D), vascular leakage (Fig. 4E), and ear thinking and swelling (Fig. 4F) in vivo*.*

### miR-let-7-5p from bMSC-EVs targeting endothelial ABL2

**Fig. 5:**
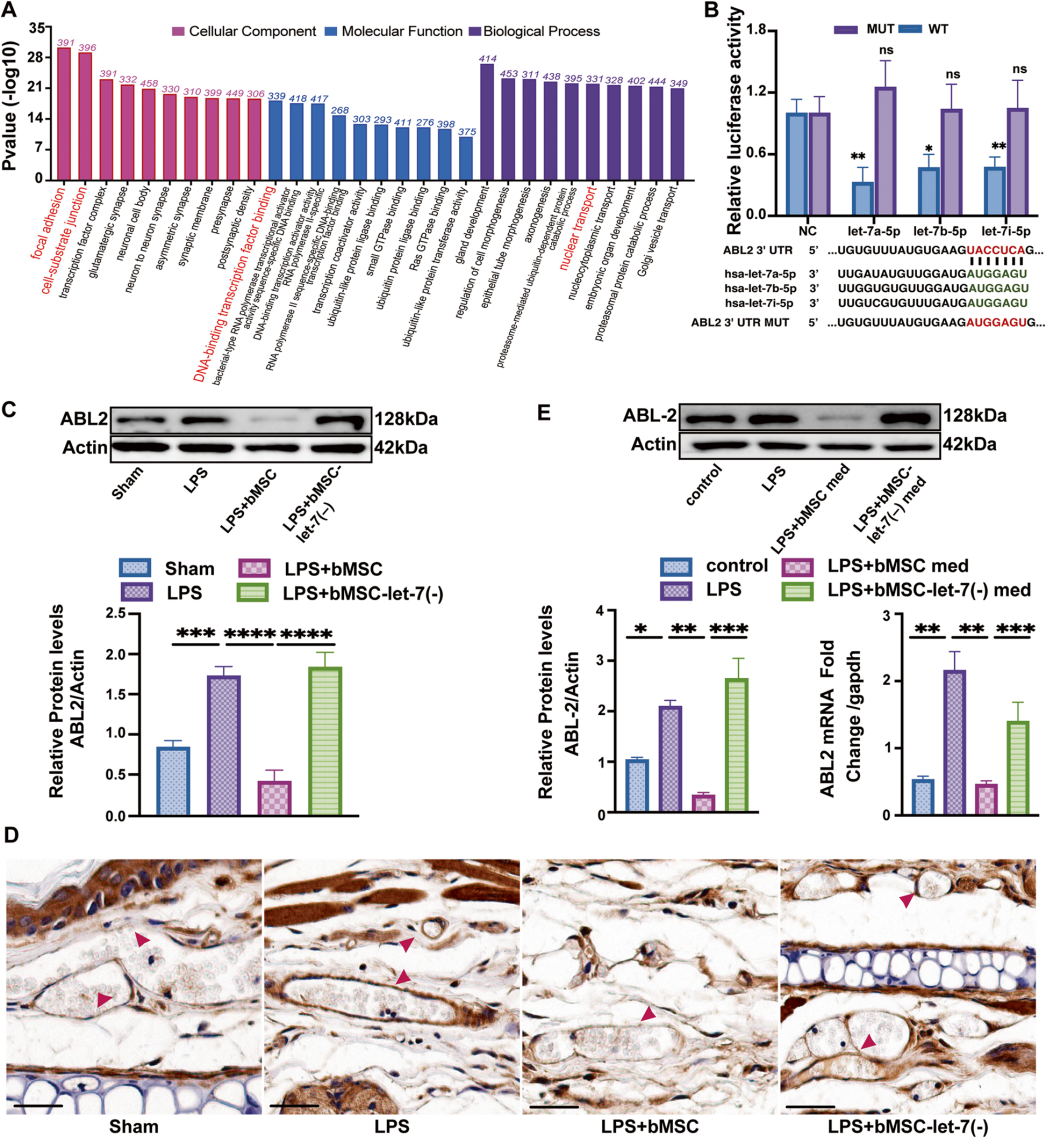
bMSC derived EVs delivered miR let-7-5p targets endothelial ABL2. **A **Highly enrichment gene ontology pathways for target genes of co-expressed miRNAs in bMSC-EVs. red label: terms of high targeting ratio, *p* < 0.001. **B **Dual-luciferase report of let-7-5p and the ABL2 gene: luciferase activity of wild-type ABL2 gene and the ABL2 gene with the 3′-UTR binding sequence of let-7a-5p, let-7b-5p, let-7i-5p sequence MUT. **C **Western blot images and quantitative analysis of the ABL2 level treated with normal bMSCs and let-7-deficient bMSCs compared to that of Actin in mice right ear tissues, (*n* = 6). **D **Immunohistochemistry images of ABL2 expression with the treatment as described in (**B**). Red arrow: ABL2 staining on ear vascular endothelium, Scale bar: 20 μm (*n* = 6). **E **Western blot images and quantitative analysis of ABL2 protein and gene expression in human umbilical vein endothelial cells (HUVECs) treated with normal bMSC med and bMSC-let-7(−) med compared with that of Actin and gapdh respectively (*n* = 3). For **B**, **C**, **D**, and **E** results were obtained at 6 h after LPS administration. Values are presented as mean ± sems; statistical analysis: *p < 0.05, **p < 0.01, ***p < 0.001, ****p < 0.0001

miRNAs interfere with protein translation by binding to the complementary nucleotide of target genes. According to the results of our sequence data, a total of 240,339 target genes of 248 co-expressed miRNAs identified from bMSC-EV samples were studied. Functional enrichment in the GO databases showed high targeting ratios at the cell matrix link cell-substrate junction and focal adhesion, DNA binding, and nuclear transportation (Fig. 5A). This suggests that miRNAs from bMSC-EV may impact EVL through target gene transcription. Studies have reported that endothelial ABL2(Cytoplasmic tyrosine kinase-2) is a kinase related to the regulation of inflammatory-related EVL [32, 33]. However, the mediating role of ABL2 in EG degradation and their relevance remains further exploration. In addition, it has not been studied whether ABL2 is a target gene of let-7-5p. Under this context, we used computational methods (RNAhybrid 2.2) and predicted that the conservative sequence of let-7a-5p, let-7b-5p, and let-7i-5p could bind with the 3′-UTR of the ABL2 gene (Fig. 5B). The putative binding sequence in ABL2-3′UTR and a mutated ABL2-3′UTR sequence were cloned for dual luciferase reporter, and the results identified a two-to-fourfold decrease in the fluorescence activity in wild-type compared with that in the MUT group after being transfected with let-7a-5p, let-7b-5p, and let-7i-5p. These data demonstrate that let-7-5p directly targets the ABL2 gene and thereby attenuates its expression (Fig. 5B).

In order to verify whether let-7-5p from bMSC-EVs would suppress endothelial ABL2 expression in vivo, we evaluated the expression of ABL2 in mice ears either treated with bMSCs or bMSCs let-7(−) after LPS stimulation. LPS elevated ABL2 protein levels in mice ear tissue, but the level decreased fourfold after bMSCs treatment whereas bMSCs let-7(−) eliminated these effects (Fig. 5C). IHC staining of ABL2 protein expression located in mice ear vascular showed similar results (Fig. 5D).

Further, identical ABL2 protein and gene expression levels were observed in HUVECs treated with bMSC med and bMSC-let-7(−) med (Fig. 5D), these results indicated that bMSC-EVs delivered let-7-5p reduced the LPS-induced ABL2 expression and verified our assumption that bMSCs suppressed EG degradation and leakage by targeting endothelial ABL2 gene.

### LPS-stimulated endothelial ABL2 to regulate p38MAPK activation contributes to EG degradation and leakage

**Fig. 6:**
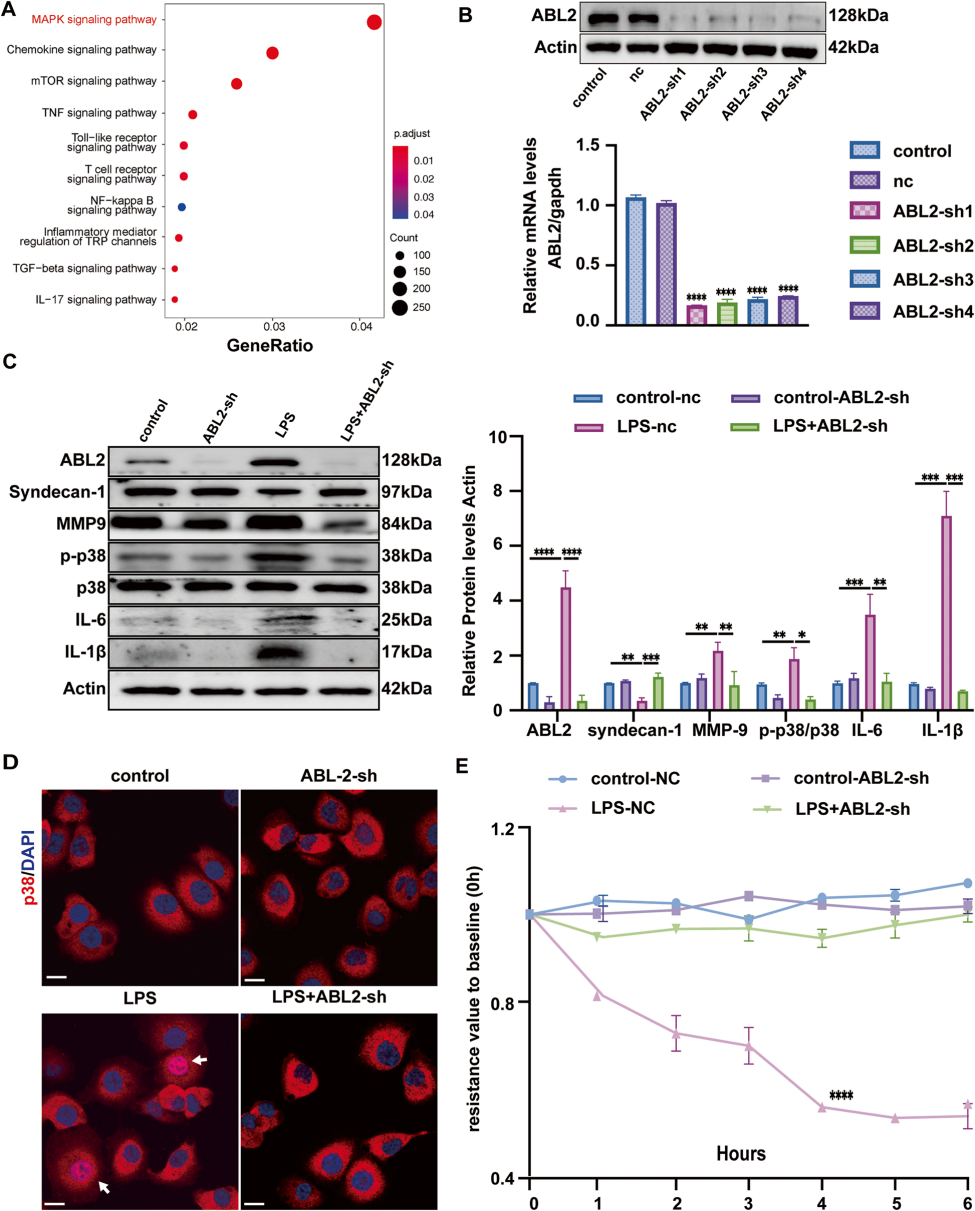
Effects of endothelial ABL2 on LPS-induced p38MAPK activation and endothelial glycocalyx (EG) degradation in vivo. **A **Highly enrichment KEGG pathways for target genes of co-expressed miRNAs in bMSC-EVs. red label: terms of high targeting ratio, *p* < 0.001. **B **Western blot images and qRT-PCR analysis of ABL2 expression compared with that of Actin and gapdh in human umbilical vein endothelial cells (HUVECs) transfected with ABL2-sh sequence using lentivirus, (*n* = 3). NC: negative control, **C **Representative Western blot images and quantitative analysis of ABL-2, syndecan-1, MMP-9, pp38/p38, IL-6, and IL-1β levels compared to that of Actin in the normal (NC) and ABL2 knockdown HUVECs (ABL2-sh) (*n* = 3). **D **Immunofluorescence images of p38 (red) expression in the NC and ABL2-sh HUVECs treated as described in (**B**) using confocal microscopy. White arrows: p38 in the nucleus; blue: DAPI. Scale bar: 50 μm (*n* = 3). **E **Transendothelial resistance value to baseline (0 h) in HUVECs treated as described in (**B**), (*n* = 3). For **C**, **D**, and **E**, results were obtained at 6 h after LPS administration. Values are presented as mean ± sems; statistical analysis: *p < 0.05, **p < 0.01, ***p < 0.001, ****p < 0.0001

The target gene function of co-expressed microRNAs from bMSC-EVs was also studied in the KEGG databases, highly targeting ratio in the inflammatory-related pathway MAPK was identified (Fig. 6A). The p38MAPK signaling pathway is known to play an important role in acute inflammation and inflammatory cytokine production. In combination with the results mentioned above, we investigate the effect of ABL2 on LPS-induced p38MAPK activation and EG degradation in vitro by silencing ABL2 in HUVECs. ABL2 knockdown HUVECs were built via lentivirus-dependent ABL2-sh sequence transfection. The Western blot and qRT-PCR results verified the inhibition efficiency of ABL2 after transfection (Fig. 6B). Protein levels of syndecan-1, MMP-9, p-p38/p38, IL-6, IL-1β in normal (NC), and ABL2 knockdown (ABL2-sh) HUVECs were evaluated after 6 h LPS administration. LPS induced a two-to-eight fold increase in the protein levels of p-p38/p38, IL-6, and IL-1β, a twofold decrease in the syndecan-1, and a twofold increase in the MMP-9 levels in HUVECs compared with those in the control NC group or LPS stimulated ABL2-sh HUVECs group (Fig. 6C). IF staining of p38 nuclear transportation was evaluated, and p38 located in the cell cytoplasm and nucleus in the LPS-stimulated NC group, whereas p38 only localized in the cell cytoplasm in the control NC and LPS + ABL2-sh group (Fig. 6D). TEER evaluation showed that LPS failed to decrease the resistance value in the ABL2-sh HUVECs compared with that in LPS + NC group. The resistance value in LPS-stimulated ABL2-sh HUVECs remained as stable as that in the control group (Fig. 6E). These results suggest that endothelial ABL2 regulates EG degradation and leakagein LPS-induced inflammation via p38-MAPK pathway activation.

## Discussion

**Fig. 7:**
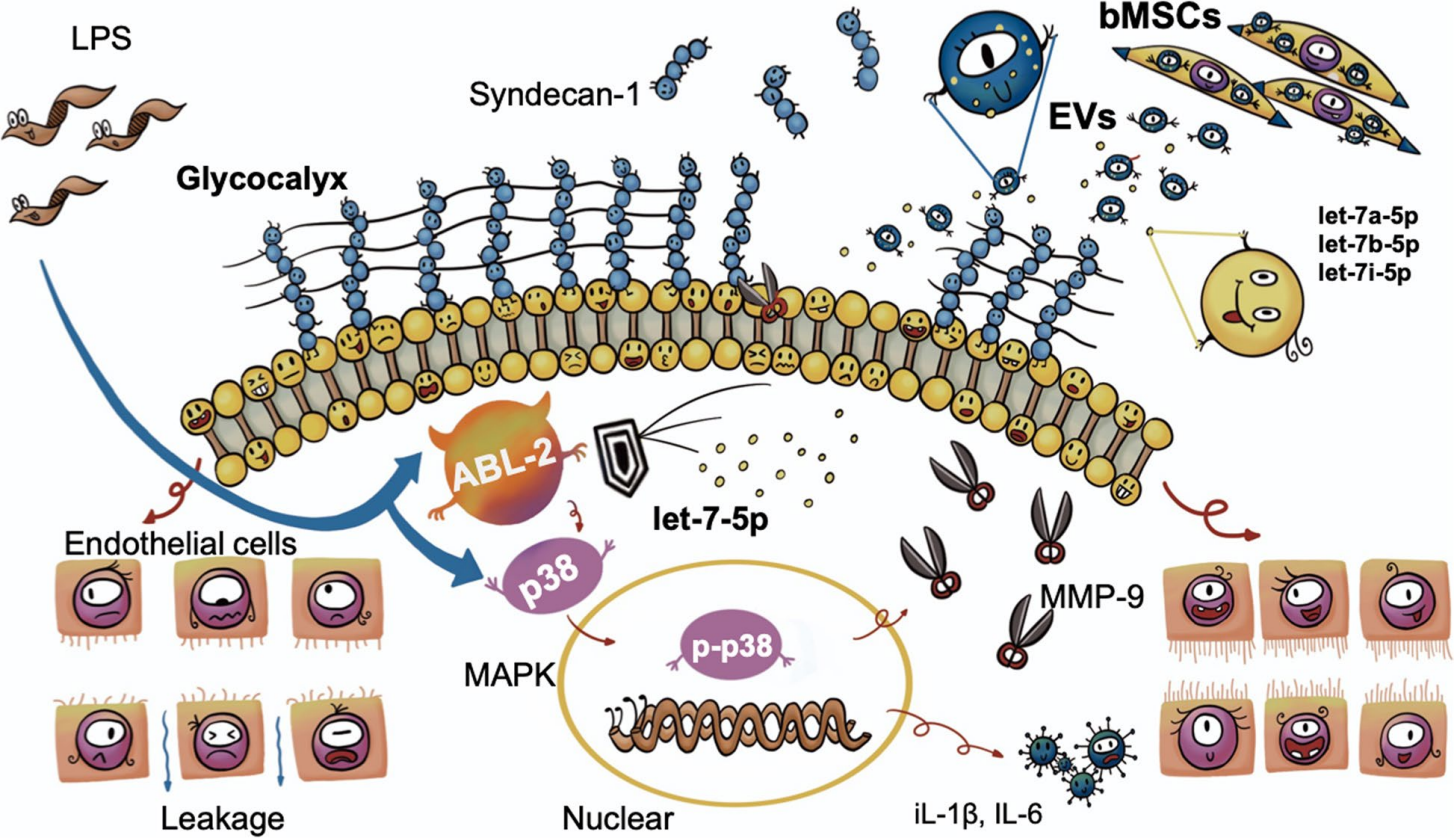
Cartoon schematic of the therapeutic mechanism of bMSC-EVs delivered miR let-7-5p targeting the endothelial ABL2. The bMSC-EVs delivered let-7-5p attenuated p38MAPK activation and reduced LPS-induced endothelial glycocalyx degradation and vascular leakage

Endothelial vascular leakage during inflammation is associated with poor clinical outcomes. Despite decades of research focused on impairment mechanisms and drug development, clinicians still lack options for pharmacological treatments that directly target EVL [34]. To the best of our knowledge, the present study is the first to clarify the therapeutic effect of bMSCs on LPS-induced EG degradation and leakage in vivo and in vitro. The underlying mechanism was further revealed as bMSC-EVs delivered miR let-7-5p targeted the endothelial ABL2 gene, inhibited the p38MAPK activation and inflammation response related EG degradation, the schematic is presented in Fig. 7.

Avoiding EVL is a key determinant for outcomes in multiple critical diseases. Most studies have struggled to identify druggable targets for EVL associated with inflammation [34]. Preclinical studies have demonstrated that EG degradation disrupts the endothelial integrity and activates ECs which induce leukocyte adhesion, cell junction separation, skeletal disruption, and further EVL [1, 35, 36]. EG is regarded as a novel promising therapeutic target. Clinically, peripheral skin (e.g., fingers and ears) is often chosen for the evaluation of endothelial injury and leakage [37], our previous study observed the LPS-induced inflammatory injury and leakage in mouse ear tissue at 6 h [29, 38]. In the present study, LPS caused the entire typical moss-like structure of EG to disappear in mice's ear vascular endothelium and leak at 6 h after LPS stimulation. It consists of a clinical trial that showed a significant reduction in sublingual gland microcirculatory glycocalyx thickness in 13 healthy volunteers after 2 h of LPS injection [39]. Despite the increased awareness of the importance of EG protection, the results of currently registered clinical studies targeting EG for treatment are uncertain. Our results showed that bMSC treatment reversed LPS-induced EG degradation in mice and HUVECs, and the therapeutic effect was accompanied by diminished tissue neutrophil infiltration or a decrease in IL-6 and IL-1β levels. These findings clarified the effectiveness of bMSCs cell therapy on LPS-induced EG degradation and implied that the related mechanism may be associated with inflammation regulation.bMSCs are known for their considerable immunomodulatory functions, a meta-analysis showed that bMSCs attenuated circulating inflammatory cytokine levels and protected against organ injury in animal studies [33]. Several laboratory studies have shown that MSCs reduce pulmonary edema by regulating calcium signaling pathways, and focal adhesion between ECs [19, 40]. The modalities and targets of inflammation modulation of bMSCs in LPS-induced EG degradation and leakage are elucidated in the present study.

- First, paracrining EVs is the effector pathway. In our study, bMSC med had an equal or even stronger effect on EG degradation than that of bMSCs in vitro; however, the therapeutic effect was lost when the EV secretion is inhibited. Our in vivo study using EV-deficient bMSCs treatment showed the same result, suggesting that EVs other than those belonging to the cellular microenvironment or metabolites affect this progress. It has been shown that the metabolic microenvironment of bMSCs may greatly affect the efficacy of cell therapy using bMSCs [41], which we hypothesize may be one of the reasons for the low therapeutic efficiency of clinical studies using MSCs in patients, for example, acidosis caused by high-carbon acidic products of metabolism leads to mitochondrial dysfunction in alveolar epithelial cells and affects the effect of transcytotic mitochondrial transfer of MSCs to alveolar macrophages [42]. Here, treatment with bMSC-EVs may be a viable approach for bMSC therapy. The bMSC-EVs maintain low immunogenicity as bMSCs and have a lower risk of differentiation and tumorigenicity. In addition, EVs can be preserved in a biocompatible medium, this would reduce the therapeutic side effects of bMSCs caused by infusion media such as fetal bovine serum and DMSO [23, 43]. Similar to our findings, bMSC med and EVs from bMSC-EVs attenuated the lung endothelial cell inflammatory response and reduced the development of pulmonary edema in vitro and in vivo [28]. In summary, our results provide encouraging evidence for bMSC-EVs therapy for EVL.
- Second, miR let-7-5p delivered by bMSC-EVs was the precise effect element. Similar to a previous study, our analysis of miRNA expression profiles in bMSC-EVs revealed the high expression of the let-7-5p family [28]. Let-7-5p expression suppressed by LPS in HUVECs was restored after treatment with bMSC med but not let-7-deficient bMSC med. Although let-7-5p has been reported to participate in the inflammatory regulation process of atherosclerosis, diabetes, and skin damage [44], the function of let-7-5p in LPS-induced EG degradation was uncertain. Our in vivo and in vitro validation by inhibiting let-7-5p in bMSC-EVs revealed its essential role in reducing LPS-induced inflammatory cytokine levels together with EG degradation and leakage. Existing studies of let-7-5p in inflammation-related diseases [45], including sepsis, have usually focused on its predictive value as a biomarker, while our results provide a basis for let-7 as a regulatory molecule of endothelial inflammation and EG degradation.
- Third, the endothelia ABL2 gene was the therapy target of inflammatory pathway p38MAPK regulation. The ABL-2 gene belongs to the ABL subfamily of cytoplasmic tyrosine kinases, which affects cell morphology and migration in tumors by phosphorylating cytoskeletal effector proteins and regulating cytoskeletal structure [46]. Kim et al. found that ABL2 inhibitors, imatinib, and nilotinib, decreased the inflammatory cytokine IL-1β level and attenuated lung edema in an LPS-induced acute lung injury mice model [47]. Our target genes enrichment of bMSC-EVs delivered miRNAs function at cellular barrier functions and the inflammatory pathway p38MAPK. The targeting and regulating between let-7-5p and ABL2 were defined and validated in vivo and in vitro by inhibiting let-7-5p in bMSC. These supported our hypothesis that ABL2 was the therapy target of let-7-5p for LPS-induced EG degradation, which is highly consistent with the inflammation regulatory role of endothelial ABL2 reported in LPS-induced EVL [32, 48]. In our study, LPS elevated protein levels of ABL2, p38MAPK phosphorylation, cytokine IL-6, and IL-1β, resulting in EG degradation and TEER decrease, knocking down ABL2 blocked these effects. In agreement with our findings, Rizzo et al. demonstrated that imatinib prevents the LPS-induced EVL in the human pulmonary artery ECs [49]. The ABL2 gene is known to be an important regulatory molecule of LPS-induced endothelial injury, yet the interrelationship between ABL2 expression, inflammatory activation, and EG degradation remains to be clarified. Our results reveal the mechanism that bMSC-EVs delivered let-7-5p targeted the endothelial ABL2 gene reduced p38MAPK related inflammatory response, providing a new therapeutic target for inflammatory-related EG degradation and leakage.

There were some limitations in this study. First, the function of ABL2 on LPS-induced p38MAPK related EG degradation was not verified in vivo. Second, taking into account the targeting effect of bMSCs, bMSC med was used instead of direct extraction of EVs in this study. Thirdly, our results demonstrated that let-7 inhibitor does not affect the expression of other lower expressed let-7 family members other than let-7a-5p, let-7b-5p and let-7i-5p (data was not given). However, due to the lack of specificity of our let-7 inhibitor, we cannot completely rule out that ABL2 could be affected by other let-7 family members, which requires further investigation. Lastly, our current study only focuses on the contribution of bMSC-EVs delivered miR let-7-5p to the treatment of EG degradation and leakage. The influence of other factors in bMSC-EVs, such as other non-coding RNA, proteins, metabolites, etc. on LPS-induced EG degradation and leakage deserves further exploration in our follow-up study. Overall, more specific and detailed mechanisms of bMSC-EVs treatment on LPS-induced EG degradation and leakage in vivo and in vitro are needed for future work.

## Conclusion

This study demonstrated the therapeutic effect of bMSCs on LPS-induced EG degradation and vascular leakage. The mechanism by which this effect was achieved involved that bMSC-EVs delivered miR let-7-5p targeting the endothelial ABL2 gene, inhibiting p38MAPK activation and inflammatory response. These findings provide a new direction for bMSC-based EG degradation and leakage treatment.

### Supplementary Information

**Additional file 1:** **Figure S1. **The scheme for the animal study. **Figure S2. **bMSCs reduce lipopolysaccharide(LPS)-induced inflammatory responses in mice and HUVECs. **Figure S3. **Enhanced effects of bMSC medium in preventing lipopolysaccharide (LPS)-induced endothelial glycocalyx (EG) degradation. **Figure S4. **miR-let-7-5p is the main effector of bMSC-EVs to suppress LPS-induced glycocalyx (EG) degradation. **Table S1. **Primary antibodies used in this study. **Table S2. **Primers used in this study.

## Data Availability

The data supporting this study’s findings are available from the corresponding author upon reasonable request.

## Abbreviations

EC: Endothelial cell
EVL: Endothelial vascular leakage
EG: Endothelial glycocalyx
EV: Extracellular vesicles
bMSC: Bone marrow mesenchymal stem cell
ARDS: Acute respiratory distress syndrome
HUVEC: Human umbilical vein endothelial cell
EB: Evans blue
LPS: Lipopolysaccharide
ELISA: Enzyme-linked immunosorbent assay
GO: Gene ontology
KEGG: Kyoto encyclopedia of genes and genomes
H&E: Hematoxylin and eosin
PBS: Phosphate-buffered saline
TEM: Transmission electron microscopy
miRNAs: MicroRNAs
TEER: Transendothelial electrical resistance
mRNA: Messenger RNA
UTR: Untranslated region
