# Supplementary material for: Bone mesenchymal stem cell extracellular vesicles delivered miR let-7-5p alleviate endothelial glycocalyx degradation and leakage via targeting ABL2
Source: Cell Commun Signal. 2023 Aug 16;21:205. doi: 10.1186/s12964-023-01229-7 (PMC10428537; doi:10.1186/s12964-023-01229-7)
Supplement: Supplementary file 2 — Additional file 1: Figure S1. The scheme for the animal study. Figure S2. bMSCs reduce lipopolysaccharide(LPS)-induced inflammatory responses in mice and HUVECs. Figure S3. Enhanced effects of bMSC medium in preventing lipopolysaccharide (LPS)-induced endothelial glycocalyx (EG) degradation. Figure S4. miR-let-7-5p is the main effector of bMSC-EVs to suppress LPS-induced glycocalyx (EG) degradation. Table S1. Primary antibodies used in this study. Table S2. Primers used in this study. [file 12964_2023_1229_MOESM1_ESM.docx]

Supplementary Materials:

**Supplementary Figures:**

Figure S1. The scheme for the animal study.

Figure S2. bMSCs reduce lipopolysaccharide(LPS)-induced inflammatory responses in mice and HUVECs.

Figure S3. Enhanced effects of bMSC medium in preventing lipopolysaccharide (LPS)-induced endothelial glycocalyx (EG) degradation.

Figure S4. miR-let-7-5p is the main effector of bMSC-EVs to suppress LPS-induced glycocalyx (EG) degradation.

**Supplementary Tables:**

Table S1. Primary antibodies used in this study

Table S2. Primers used in this study

Figure S1. The scheme for the animal study. A. Lipopolysaccharide(LPS) induced mice ear vascular leakage model. 20 μl of LPS (125 μg/μl) was smeared on mice's right ears after pricking in the LPS groups, normal saline was used as a placebo for the control groups and the left ears. Arrows: pricked regions, orange: inner side; black: outer side. B. Bone marrow mesenchymal stem cells (bMSCs) therapy model and Evans Blue (EB) leakage evaluation. 100 μl resuspended primary bMSCs (2×10^7^ cells) and 100 μl 2% Evans Blue were tail injected respectively at 2h and 4h after modeling. At the 6h the mice were sacrificed, and samples were collected for further studies.


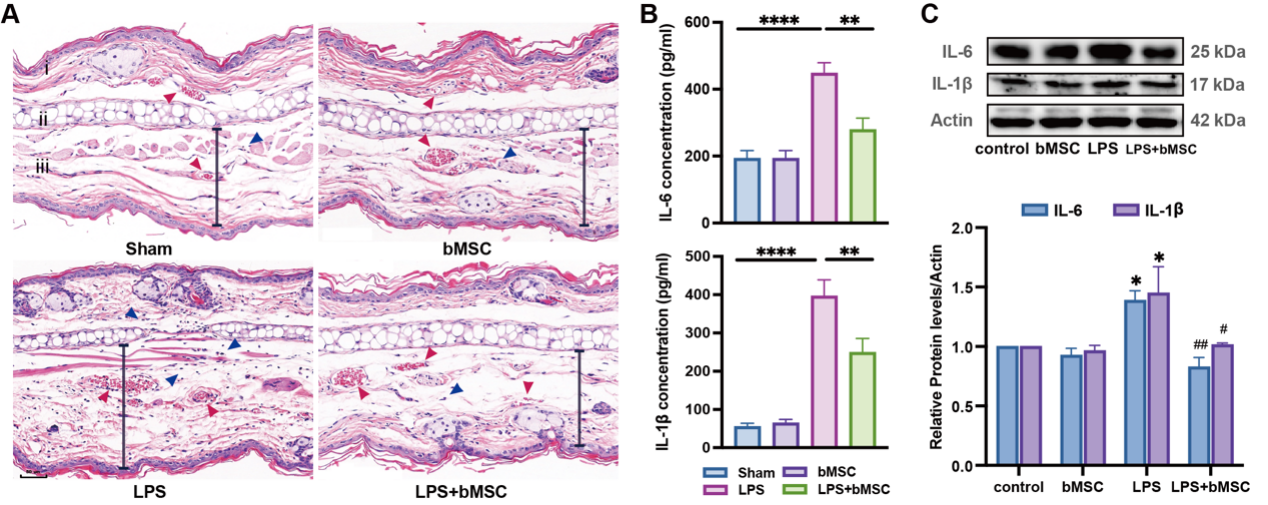


Figure S2. bMSCs reduce lipopolysaccharide(LPS)-induced inflammatory responses in mice and HUVECs. A. Hematoxylin and eosin staining images of the mice right ear tissue. i, skin; ii, cartilage; and iii, subcutaneous tissue. Black arrows: ear vascular endothelium, blue arrows: neutrophil infiltration, red arrows: red blood cells indicating hemorrhage, and gray line: tissue thickness indicating edema. Scale bar: 50 µm. B. Enzyme-linked immunosorbent assay analysis of IL-6 and IL-1β levels in right ear tissue (n = 6). C. Western blot images and quantitative analysis of IL-6 and IL-1β levels in HUVECs compared to that of Actin. All results were obtained at 6 h after LPS administration. Values are presented as mean ± sems. Statistical analysis: *p < 0.05, **p < 0.01, ***p < 0.001, ****p < 0.0001, ^#^p < 0.05, ^##^p < 0.01, ^###^p < 0.001, ^####^p < 0.0001 compared with the LPS group.


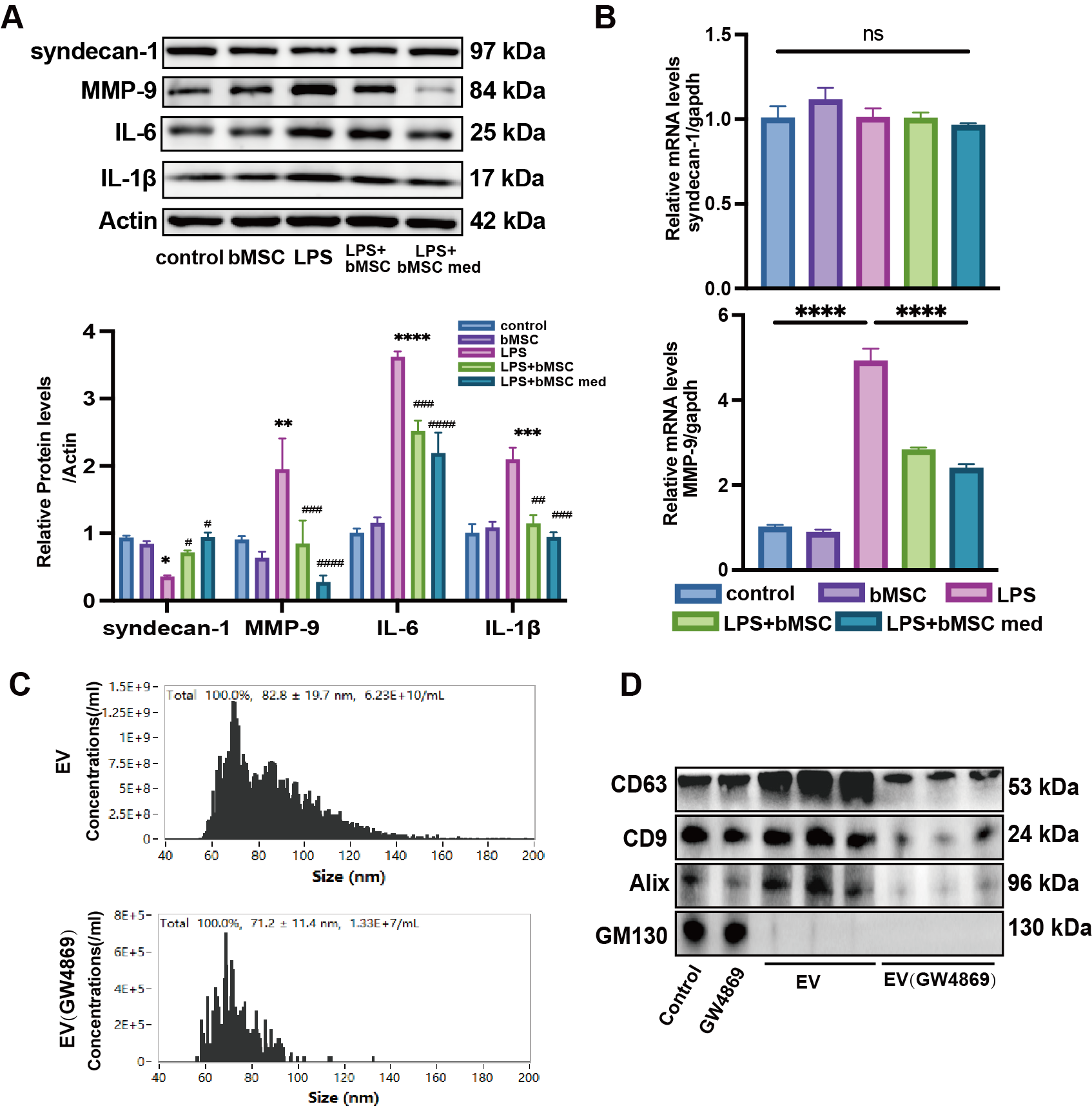


Figure S3. Enhanced effects of bMSC medium in preventing lipopolysaccharide (LPS)-induced endothelial glycocalyx (EG) degradation. A. Western blot images and quantitative analysis of syndecan-1, MMP-9, IL-6, and IL-1β levels compared to that of Actin in HUVECs treated with bMSCs or bMSC med (n = 3). B. qRT-PCR analysis for fold changes of syndecan-1 and MMP-9 gene expression to gapdh in HUVECs (n = 3). All results were obtained at 6 h after LPS administration. Values are presented as mean ± sems. Statistical analysis: *p < 0.05, **p < 0.01, ***p < 0.001, ****p < 0.0001compared with the control group, ^#^p < 0.05, ^##^p < 0.01, ^###^p < 0.001, ^####^p < 0.0001 compared with the LPS group. C. Concentration of the EVs extracted from bMSC med and EVs extracted from GW4869 pre-treated bMSC med measured by Nano Flow Cytometer. D. Western blot images of CD63, CD9, Alix, and GM130 proteins of bMSC, GW4869 pre-treated bMSCs, EVs extracted from bMSC med, and EVs extracted from GW4869 pre-treated bMSC med (n=3).


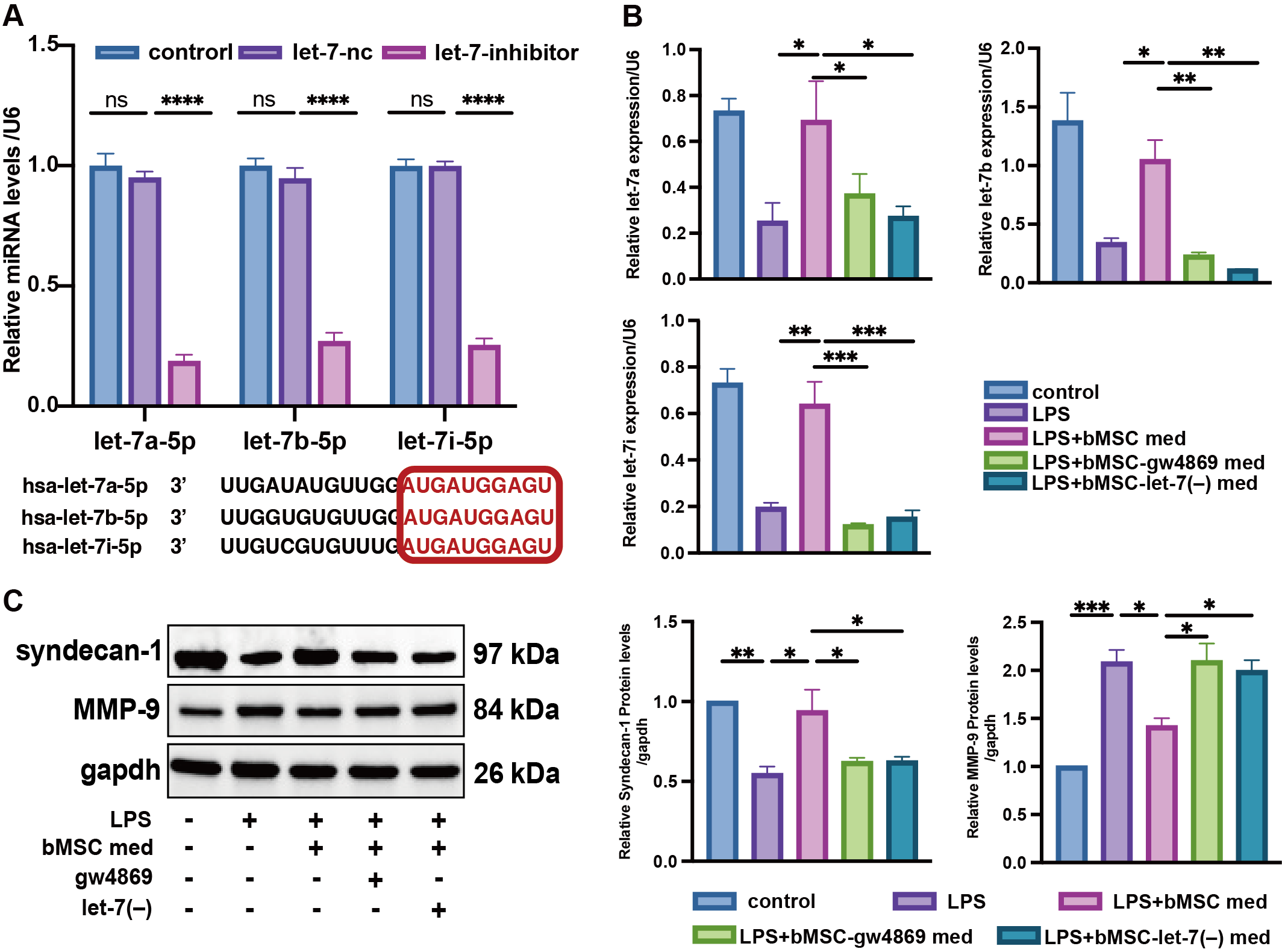


Figure S4. miR-let-7-5p is the main effector of bMSC-EVs to suppress LPS-induced glycocalyx (EG) degradation. A.qRT-PCR analysis for fold changes of let-7a-5p, let-7b-5p, and let-7i-5p expression levels in bMSCs after transfection with negative control and let-7 inhibitor. (n = 3). B. qRT-PCR analysis of let-7a-5p, let-7b-5p, let-7i-5p expression in HUVECs treated with bMSC med, bMSC- med, and bMSC-let-7(–) med (n = 3). C. Western blot images and quantitative analysis of syndecan-1, MMP-9 levels compared to that of Actin in HUVECs with the treatment described in (B) (n = 3). For B and C, results were obtained at 6 h after LPS administration. Values are presented as mean ± sems. Statistical analysis: *p < 0.05, **p < 0.01, ***p < 0.001, ****p < 0.0001.

Table S1. Primary antibodies used in this study

| Antigens | Manufacturer | Catalog Number | Applications |
| --- | --- | --- | --- |
| Syndecan-1 | ABclonal, China | A4174 | 1:1000 FOR WB |
| Syndecan-1 | Abcam, UK | ab128936 | 1:200 FOR IF |
| ABL2 | ABclonal, China | A19628 | 1:1000 FORWB 1:200 FOR IHC |
| MMP-9 | Affinity, USA | AF5228 | 1:1000 FOR WB |
| CD31 | Abcam, UK) | ab24590 | 1:200 FOR IF |
| GAPDH | Cell Signaling Technology, USA | # 5174S | 1:5000 FOR WB |
| phospho-p38 | Cell Signaling Technology, USA | #4511 | 1:1000 FOR WB |
| p38 | Cell Signaling Technology, USA) | #8690 | 1:1000 FOR WB  1:200 FOR IF |
| Actin | Cell Signaling Technology | #4970, | 1:2000 FOR WB |
| IL-6 | ABclonal, China | A0286 | 1:1000 FOR WB |
| IL-1β | Cell Signaling Technology, USA | #12703 | 1:1000 FOR WB |
| Alexa Fluor 594 | Abcam, UK | ab150088 | 1:200 FOR IF |
| Alexa Fluor 594 | Abcam, UK | ab150116 | 1:200 FOR IF |
| Alexa Fluor 488 | Abcam, UK | ab150077 | 1:200 FOR IF |

Table S2. Primers used in this study

| Gene/miRNA | Forward primer (5′–3′) | Reverse primer (5′–3′) | RT primer |
| --- | --- | --- | --- |
| H-Syndecan-1 | CTGCCGCAAATTGTGGCTAC | TGAGCCGGAGAAGTTGTCAGA |  |
| H-ABL2 | GTTGAACCCCAGGCACTAAAT | CAACGAAGAGATTAGGGTCACTC |  |
| H-MMP-9 | TGTACCGCTATGGTTACACTCG | GGCAGGGACAGTTGCTTCT |  |
| H-GAPDH | GGAGCGAGATCCCTCCAAAAT | GGCTGTTGTCATACTTCTCATGG |  |
| hsa-let-7a-5p | GCTGCGTGAGGTAGTAGGTTGT | AGTGCAGGGTCCGAGGTATT | GTCGTATCCAGTGCAGGGTCCGAGGTATTCGCACTGGATACGACAACTAT |
| hsa-let-7b-5p | GCTGCGTGAGGTAGTAGGTTGT | AGTGCAGGGTCCGAGGTATT | GTCGTATCCAGTGCAGGGTCCGAGGTATTCGCACTGGATACGACAACCAC |
| hsa-let-7i-5p | CGTCGCGTGAGGTAGTAGTTTGT | AGTGCAGGGTCCGAGGTATT | GTCGTATCCAGTGCAGGGTCCGAGGTATTCGCACTGGATACGACAACAGC |
| U6 | CTCGCTTCGGCAGCACA | AACGCTTCACGAATTTGCGT | GTCGTATCCAGTGCAGGGTCCGAGGTATTCGCACTGGATACGACAAAATATGGA |

RT: reverse transcriptase
